# Mixoma Ventricular Izquierdo

**DOI:** 10.47487/apcyccv.v1i1.13

**Published:** 2020-03-30

**Authors:** Gerber Polo, Julio Castillo, Manuel Dávila

**Affiliations:** 1 Médico residente de Cirugía de Tórax y Cardiovascular - Instituto Nacional Cardiovascular INCOR. Lima, Perú. Instituto Nacional Cardiovascular INCOR Lima Perú; 2 Servicio de Cirugía Cardiovascular Adulto - Instituto Nacional Cardiovascular INCOR. Lima, Perú. Servicio de Cirugía Cardiovascular Adulto Instituto Nacional Cardiovascular INCOR Lima Perú

**Keywords:** myxoma, tumoración de ventrículo izquierdo, myxoma, left ventricular tumor

## Abstract

El mixoma es un tumor cardíaco primario de poca prevalencia en la población, con predilección por la aurícula izquierda seguido por la aurícula derecha. La localización en los ventrículos es infrecuente y la sintomatología varía según el lugar de origen. Sus complicaciones asociadas pueden tratarse o evitarse con la resección quirúrgica oportuna.

Presentamos el caso de una mujer de 15 años de edad, sintomática, con una masa en el ventrículo izquierdo. La masa fue resecada quirúrgicamente, y el estudio histopatológico confirmó que se trataba de un mixoma.

## Abstract (en)

Myxoma is a primary cardiac tumor of low prevalence in the population, with a predilection for the left atrium followed by the right atrium. The location in the ventricles is infrequent and the symptoms depends of the place of origin. Its associated complications and its particular form of debut can be avoided with timely surgical resection.

We present the case of a 15-year-old symptomatic woman with a mass in the left ventricle. The mass was surgically resected, and the histopathological study confirmed a myxoma.

La prevalencia de los tumores cardíacos primarios se encuentra entre 0,01 a 0,03% en la población general (basado en diversas series de autopsias).^1^^,^^2^ De estos tumores, los mixomas constituyen los más frecuentes y representan más del 75-80% de todas las neoplasias cardíacas benignas, derivan de células mesenquimales multipotenciales situadas en el endocardio y se pueden originar en cualquier cámara cardiaca.^3^

La localización más frecuente de los mixomas es en la aurícula izquierda (80%) seguido de la aurícula derecha (20%), el ventrículo derecho (3%) y el ventrículo izquierdo (3%).^1^ El cuadro clínico depende de esta localización, por lo que puede variar desde ser asintomático hasta tener presentaciones clínicas graves como síncope, falla cardiaca, embolia pulmonar y/o sistémica e incluso la muerte.^4^ Si el tumor afecta el tejido de conducción puede ocasionar arritmias cardiacas auriculares (fibrilación, flutter o taquicardia supraventricular) o del tipo ventricular (taquicardia ventricular o fibrilación ventricular) e inclusive pueden producir bloqueo auriculoventricular con posibilidad de muerte súbita.^5^

El diagnóstico se realiza principalmente mediante la ecocardiografía, complementada con la tomografía y resonancia magnética, las cuales proporcionan información muy útil para caracterizar la naturaleza y extensión del tumor, así como para planificar la resección quirúrgica.^3^

Debido a su naturaleza móvil, los mixomas pueden prolapsar y producir embolias. Aquellos ubicados en el ventrículo izquierdo tienen el mayor riesgo de morbi-mortalidad por embolización a la circulación sistémica u oclusión parcial o completa del tracto de salida del ventrículo izquierdo y de la circulación coronaria,^6^ por lo que al diagnosticarlo en muchos casos requiere de cirugía de urgencia.^7^

Presentamos el caso de una paciente portadora de un tumor ventricular izquierdo, compatible con mixoma, con sintomatología asociada, y que fue programada para cirugía de urgencia. 

## Descripción del Caso

**Figura 1:**
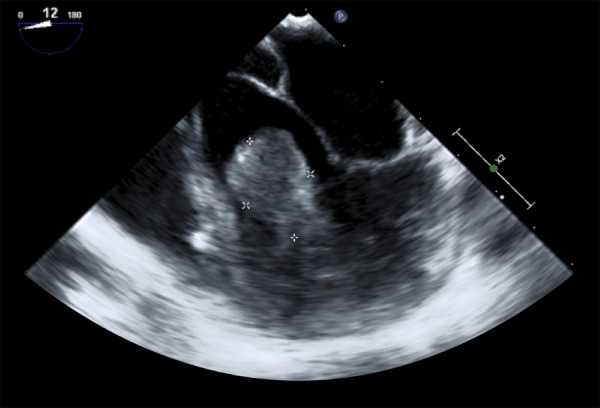
Ecografía transesofágica muestra tumor de ventrículo izquierdo de 22 x 13 mm. que no obstruye significativamente el tracto de salida del ventrículo izquierdo.

**Figura 2:**
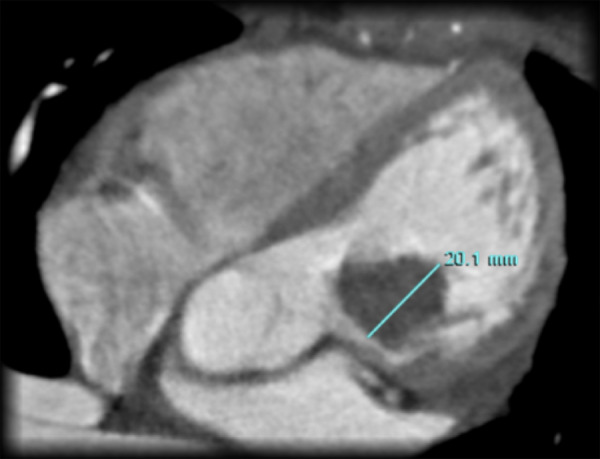
Angiotomografía que demuestra ausencia de contraste en ventrículo izquierdo y tracto de salida del mismo, correspondiente a tumoración ventricular.

Paciente mujer de 15 años de edad, con antecedente de episodios de arritmia cardíaca no especificada desde hace 3 años, en tratamiento con verapamilo y propanolol (el electrocardiograma de ingreso a nuestra institución mostraba ritmo sinusal). Presentaba desde hace 2 meses palpitaciones y dolor torácico esporádico; se le realiza ecocardiografía (Figura 1) y posteriormente una angiotomografía (Figura 2), observándose una tumoración móvil, ovalada, de 21 x 14 mm en ventrículo izquierdo (VI), con pedículo adherido al borde superior de VI, que no producía obstrucción significativa del tracto de salida del VI, con características sugestivas de mixoma. Posterior al diagnóstico imagenológico, se le indicó tratamiento quirúrgico de urgencia debido al riesgo que tienen este tipo de tumoraciones de embolizar a la circulación sistémica.

**Figura 3:**
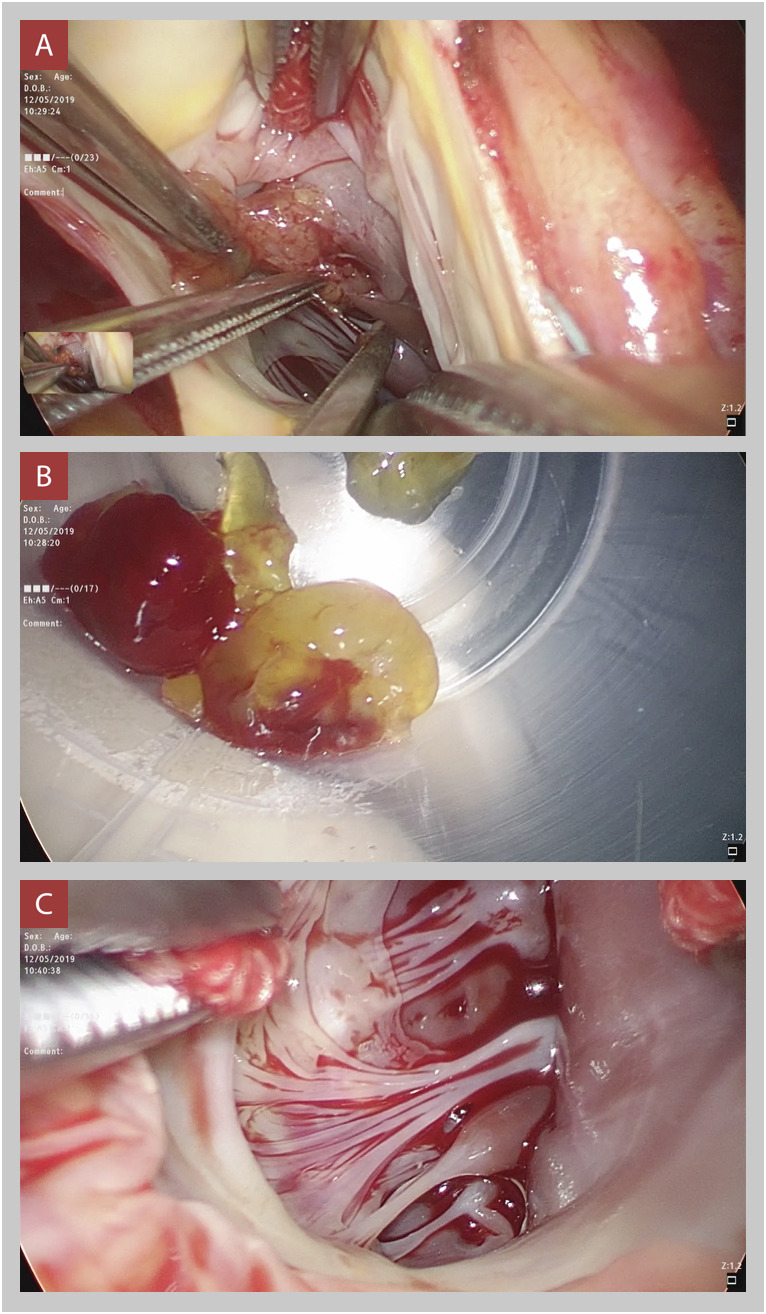
A. Exéresis de tumoración, se observa dependencia de músculo papilar en cara anterior de ventrículo izquierdo. B. Pieza quirúrgica, características morfológicas compatibles con mixoma. C. Se muestra el tracto de salida del ventrículo izquierdo y parte de la cavidad ventricular, sin evidencia de tumoración residual.

**Figura 4:**
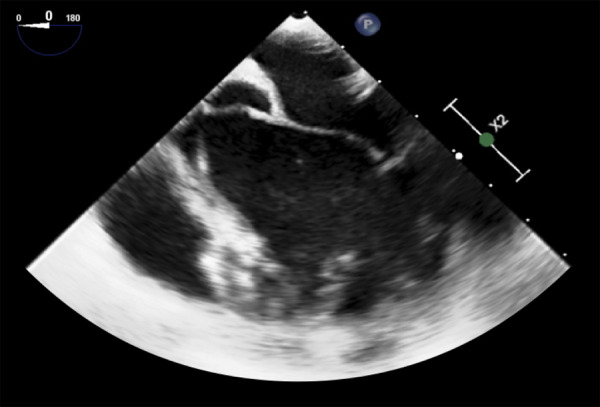
Control post cirugía: Ecocardiografía transesofágica muestra ausencia de tumoración residual en ventrículo izquierdo.

Se realizó una esternotomía media con canulación central atrio-cava, el abordaje fue trans-aórtico, y se exploró directamente el tracto de salida del ventrículo izquierdo y la cavidad ventricular. Se evidenció una tumoración dependiente de músculo papilar en cara anterior de VI de aproximadamente 20 x 20 mm. (Figura 3A) Se procedió a realizar la exéresis completa de la tumoración video asistida; obteniéndose una tumoración de aspecto gelatinoso, de consistencia blanda y con superficie externa brillante. (Figura 3B) Posteriormente se exploró la cavidad ventricular confirmándose la extracción completa del tumor. (Figura 3C) El tiempo total de circulación extracorpórea (CEC) fue de 67 minutos y de clampaje aórtico, 40 minutos. En la ecografía transesofágica post CEC (circulación extracorpórea) se observó la cavidad ventricular y el tracto de salida de VI sin evidencia de tumoración residual. (Figura 4)

En el postoperatorio, fue extubada a las 6 horas, con una estancia en la unidad de cuidado intensivo de 48 horas. Fue dada de alta a los 6 días de su ingreso con un control ecocardiográfico que mostró ausencia de tumoración residual, función biventricular normal, y sin patología valvular. El informe anatomopatológico indicó que la tumoración resecada del VI correspondía a un mixoma.

## Discusión

El mixoma cardíaco es un tumor primario benigno poco frecuente en la población. Algunos estudios nacionales reportan datos sobre las características clínicas de los pacientes con tumoraciones cardiacas atendidos en los diferentes centros, mas no sobre la incidencia o prevalencia. 

La localización más frecuente para los mixomas es la aurícula izquierda; en una cohorte, 14 de 18 se alojaron en la aurícula izquierda (78% de los casos),^4^ una frecuencia un poco mayor se indica en un reporte de casos de nuestro pais, en la cual 22 de 26 mixomas se alojaron en la mencionada cavidad (84%).^8^

Nuestro reporte trata sobre el caso de una paciente con tumor en cavidad ventricular izquierda. Esta localización es menos frecuente, y se ha informado una tasa del 2.5% al 4 % en reportes internacionales^9^ y de 3.8% según un estudio nacional.^8^

Su presentación es más común entre la cuarta y la sexta décadas de la vida;^10^ sin embargo, la edad de nuestra paciente fue de 15 años, lo que nos muestra su distribución muy amplia con un rango entre 1 a 86 años según una serie.^4^ Nuestra paciente fue de sexo femenino que es el predominante en los mixomas cardíacos, con una preponderancia femenina de 2:1.^11^

Los pacientes con un mixoma cardíaco generalmente son asintomáticos o tienen síntomas inespecíficos que dependen de la ubicación, el tamaño, la movilidad y la friabilidad de la masa. La mayoría de los mixomas de origen ventricular presentan signos y síntomas como síncope, tromboembolismo, arritmias, insuficiencia cardíaca congestiva y muerte súbita.^12^ Nuestra paciente tenía como antecedente una arritmia cardiaca no especificada desde hace 3 años (sin otra sintomatología asociada en ese entonces) lo cual nos hace pensar en que esta arritmia podría deberse al mixoma ventricular. 

En la actualidad, la ecocardiografía transtorácica es la técnica de imagen primaria para el diagnóstico, por su disponibilidad, bajo costo y no invasividad, siendo la tasa de detección del 95,2%. Proporciona información importante, como el sitio del tumor, el tamaño, el número, el pedículo y los cambios hemodinámicos, así como la función ventricular y la función valvular.^13^ La tomografía computarizada y la resonancia magnética permiten evaluar la relación entre el tumor cardíaco y las estructuras intracardíacas o extra cardiacas adyacentes, y pueden ayudar al diagnóstico diferencial.^14^

El tratamiento de elección es la resección quirúrgica y la esternotomía media es el enfoque convencional para la resección de estos tumores.^11^ En la paciente, luego de la esternotomía media, se utilizó un abordaje trans-aórtico para así evitar una ventriculotomía izquierda y sus posibles complicaciones.^15^La resección del mixoma no requiere de la extirpación completa de la pared ventricular, porque no se han observado recidivas cuando se realizan resecciones más limitadas.^7^^)^ Como resultado, se logró la escisión quirúrgica completa del tumor sin complicaciones inmediatas ni mediatas hasta los 2 meses de seguimiento. 

El pronóstico de los tumores cardíacos benignos primarios es bueno siempre que se logre una resección tumoral completa. El resultado quirúrgico para el mixoma cardíaco es generalmente bueno, con una tasa de supervivencia a 20 años del 85%.^15^^)^ Las recurrencias tardías han sido reportadas en 0,4-5% de los pacientes tratados quirúrgicamente de 3 meses a 22 años después de la cirugía.^1^

## Conclusión

El mixoma ventricular izquierdo es un raro tumor, su clínica asociada es variable y puede ir desde ser asintomático hasta presentar muerte súbita. Dichas complicaciones pueden tratarse o evitarse con la resección quirúrgica oportuna.

## Financiación:

Ninguna
